# Is a rare *CXCL8* gene variant a new possible cause or course factor of inflammatory bowel disease?

**DOI:** 10.3389/fimmu.2025.1562618

**Published:** 2025-03-19

**Authors:** Marcin Gabryel, Oliwia Zakerska-Banaszak, Karolina Ladziak, Katarzyna Anna Hubert, Alina Baturo, Joanna Suszynska-Zajczyk, Magdalena Hryhorowicz, Agnieszka Dobrowolska, Marzena Skrzypczak-Zielinska

**Affiliations:** ^1^ Department of Gastroenterology, Dietetics and Internal Medicine, Poznan University of Medical Sciences, Poznan, Poland; ^2^ Institute of Human Genetics, Polish Academy of Sciences, Poznan, Poland; ^3^ Department of Biochemistry and Biotechnology, Poznan University of Life Sciences, Poznan, Poland

**Keywords:** CXCL8 gene, inflammatory bowel disease (IBD), inflammation, interleukin 8, genetic variants, Crohn’s disease, colitis ulcerosa

## Abstract

**Introduction:**

The pathogenesis of inflammatory bowel diseases (IBD) involves genetic, environmental, immunological, and microbial factors; however, it remains unclear. Pro-inflammatory interleukin 8 (IL-8), encoded by the *CXCL8* gene, assumes a crucial chemotactic role in leukocyte migration.

**Methods:**

This study aimed to investigate whether an association exists between IBD and two *CXCL8* variants, namely, c.-251A>T (rs4073) and c.91G>T (rs188378669), and IL-8 concentration. We analyzed the distribution of both variants among 353 Polish IBD patients and 200 population subjects using pyrosequencing, competitive allele-specific PCR and Sanger sequencing.

**Results:**

The c.91T stop-gained allele was significantly more frequent in IBD patients (2.12%) than in controls (0.25%) (*p* = 0.0121), while the c.-251T allele frequencies were similar (54% vs. 51.5%, *p* = 0.4955). Serum IL-8 concentrations, measured using ELISA, were higher in IBD patients with the c.91 GG genotype compared to healthy controls (mean, 70.02 vs. 51.5 pg/ml, *p*<0.01) and patients with c.91 GT (mean, 61.73 pg/ml). Moreover, clinical data indicated that carriers of the c.91T variant need more often corticosteroids and surgical treatment of the disease than GG homozygous IBD patients.

**Conclusion:**

This suggest that the *CXCL8* c.91T allele may influence IBD manifestation and the course of the disorders in Polish patients, potentially serving as a novel target for future studies and therapeutic approaches.

## Graphical Abstract

Graphical abstract of the study. Created in BioRender. Skrzypczak-zielinska, M. (2025) https://BioRender.com/o10i655.
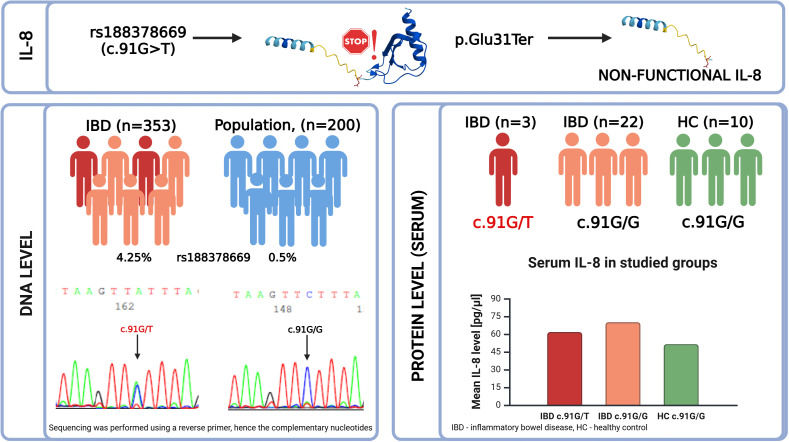

## 1 Introduction

Inflammatory bowel diseases (IBDs), including Crohn’s disease (CD) and ulcerative colitis (UC), are a group of chronic inflammatory diseases of the gastrointestinal (GI) tract. These conditions are incurable and characterized by alternating periods of exacerbation and remission. IBD is often referred to as a civilization disease due to its higher prevalence in populations of highly developed countries (1). Unfortunately, the etiology of IBD remains poorly understood, contributing to the lack of causal treatments. Pathogenesis of IBD is complex; many factors, such as genetic, environmental, immunological, and microbial, influence their development (2, 3). However, ongoing research still aims to fully elucidate the molecular pathomechanisms underlying these diseases.

Global studies indicate that factors regulating the inflammatory process within the body are critical contributors to the development of various inflammatory diseases, including IBD (4). Interleukin 8 (IL-8, CXCL8) encoded by the *CXCL8* gene is one of the first and most intensively studied chemokines and can be released by different cell types, including monocytes, endothelial cells, intestinal epithelial cells, and T lymphocytes (5). Activation of IL-8 by cytokines (such as interleukins 1 and 6, interferon-γ, tumor necrosis factor-alpha, and even IL-8 itself), bacterial molecules, lipopolysaccharide, hypoxia, and other cellular stresses increase its level several folds. The primary function of IL-8 is to activate inflammatory cells and enhance the immune response by acting as a chemotactic factor. This property enables the migration of leukocytes, mainly neutrophils, from peripheral blood to inflamed tissues. IL-8 binds to G-protein-coupled receptors, CXCR1 and CXCR2, present on the surface of neutrophils and triggers cellular signaling leading to neutrophil activation. Consequently, IL-8 plays a pivotal role in the innate immune response (6).

The *CXCL8* gene (MIM 146930) encoding IL-8 is implicated in the etiology of several chronic inflammatory diseases, including asthma, rheumatoid arthritis, skin inflammation, periodontitis, and cancer (7–9). Sequence variants of the *CXCL8* gene are responsible for developing those disorders via two mechanisms. The first mechanism involves the *CXCL8* gene expression caused by polymorphisms in the promoter region that lead to alteration in transcription factor binding. Changes in *CXCL8* expression levels influence the intensity of the pro-inflammatory response and are often associated with distinct disease phenotypes. The second mechanism involves structural modifications in the receptor binding sites in the IL-8 protein, which can alter receptor affinity (10, 11).

More than 700 SNPs (NCBI dbSNP) in the *CXCL8* gene have been identified, focusing on those located in the promoter and protein-coding regions. The c.-251A>T (rs4073) variant is situated in the *CXCL8* gene at the transcription factor NF-KB binding site and partially at the transcription enhancer-binding position (12). The NF-κB activation pathway has garnered significant interest from scientists due to its role in *CXCL8* transcription (13). Both *in vitro* and *in vivo* research revealed that allele c.-251T is associated with an increased level of IL-8 (14). Certain studies have demonstrated inappropriate activation of this pathway in gastrointestinal conditions (15). The variant allele (T) frequency in the European population is 57.9% (1000 Genomes Project Phase 3).

Meanwhile, variants in the IL-8 protein-coding region can significantly affect the activity and structure, which disturbs the course of the inflammatory reaction cascade. One notable substitution is c.91G>T (rs188378669), which results in the premature termination of the IL-8 protein due to an amino acid change (p.Glu31Ter) (12). The frequency of this rare variant is <1% (1000 Genomes Project Phase 3). However, in our previous investigation on genetic factors determining the response to glucocorticoids, we observed in our study group, i.e., among patients with IBD, an over eightfold higher frequency of this variant compared to the European population (16). This finding prompted us to study a larger IBD patient cohort to determine if either of these two variants in the *CXCL8* gene may be associated with the manifestation of IBD and the inflammatory process cascade.

## 2 Materials and methods

### 2.1 Patients and samples

A group of 353 IBD patients (189 with CD and 164 with UC) under the care of the University Clinical Hospital in Poznan, the Department of Gastroenterology, Dietetics, and Internal Medicine, along with 200 subjects (104 women and 96 men) from the Polish population, were randomly enrolled to this study. IBD patients’ middle age was 46.7 ± 17.1 years, and for the population, 43.4 ± 15.2 years. CD and UC were diagnosed based on the clinical, endoscopic, and histological data. All participants provided written informed consent. To conduct the study at the protein level, we used serum material from available three IBD patients with the DNA substitution, 23 IBD patients without the genetic change, and 10 healthy individuals in whom IBD was excluded and who did not have the tested variant. The study was approved by the local Ethics Committee of Poznan University of Medical Sciences (resolution nos. 436/13 and 466/20). All experiments were performed in accordance with the principles of the 1964 Declaration of Helsinki and its subsequent amendments.

### 2.2 DNA isolation

Genomic DNA was isolated from peripheral blood samples of all individuals using the guanidine isothiocyanate method. Blood was collected into 5-ml EDTA S-Monovette tubes (Sarstedt, Numbrecht, Germany). The resulting DNA samples were stored at 4°C in TE buffer (10 mM Tris–HCl, 0.1 mM EDTA, pH 8) until use.

### 2.3 *CXCL8* locus amplification

The amplification of *CXCL8* gene fragment containing promoter region with variant c.-251A>T was carried out in a total volume of 30 μl using 0.75 U of FIREPol^®^ DNA Polymerase, 2.5 μl 10× buffer, 2.0 μl dNTP mix (2.5 mM each dNTP), 1.5 mM MgCl2 solution, 80 ng DNA, and 0.2 μM of each primer (forward, 5′ATCTTGTTCTAACACCTGCCACTC3′, and biotinylated reverse, 5′AAGCTCCACAATTTGGTGAATTA3 ′). Amplification involved 50 cycles at 95°C for 30 s, 58°C for 30 s, and 72°C for 60 s. All reagents were obtained from Solis BioDyne (Tartu, Estonia). The PCR products with a length of 112 bp were checked in 1.7% agarose gel electrophoresis.

### 2.4 Pyrosequencing and competitive allele-specific PCR

Genotyping of rs4073 locus in *CXCL8* gene by pyrosequencing was performed with a sequencing primer (5′TAGAAATAAAAAAGCATACA3′) using the PSQ™ 96MA system (Qiagen GmbH, Hilden, Germany) and PyroMark™ Gold Q96 Reagents (Qiagen) as described by the manufacturer.

For genotyping of the rs188378669 (c.91G>T; p.Glu31Ter) in the *CXCL8* gene, competitive allele-specific PCR (KASP) was used. The reaction mix contained one forward primer, two competitive reverse primers (one complementary to the G allele labeled with FAM and the other complementary to the T allele labeled with HEX), master mix, genomic DNA, and water. The PCR reaction was carried out on CFX Connect Real-Time PCR Detection System (Bio-Rad) according to the manufacturer’s instructions. Fluorescent signals were detected using Bio-Rad CFX Manager. Results were grouped based on genotype.

### 2.5 Sanger sequencing

Samples grouped using the KASP method were randomly selected for sequencing to confirm the genotype by Sanger sequencing. Sequencing was performed using BigDye Terminator v3.1 Cycle Sequencing Kit (Thermo Fisher Scientific, Waltham, MA, USA) on the Applied Biosystems 3500 Series Genetic Analyzer. For this purpose, the following primers were used: forward, 5′ATCACTTTTTCCCCCAACAG3′ and reverse 5′CCTAACACCTGGAACTTTCCTAAA3′ for amplification of 246 bp product.

### 2.6 ELISA assay

The IL-8 concentration in serum was analyzed using an enzyme-linked immunosorbent assay (ELISA), Human IL-8 ELISA Kit (Diaclone, cat. no. 950.050.192), according to the manufacturer’s protocol. The absorbance was measured at 450 nm wavelength using an EL-808 scanner spectrometer (BioTek Instruments Inc., USA). The test sensitivity was 12.3 pg/ml, and the detection range was 31.25–1,000 pg/ml.

### 2.7 *In silico* prediction of variant consequence and structure-based function

To evaluate the potential consequences of the rs188378669 (c.91G>T) variant, a comprehensive *in silico* analysis was conducted using multiple bioinformatic tools and databases. To analyze the structural characteristics of interleukin-8 (IL-8) protein, the AlphaFold AI (17, 18) system developed by Google DeepMind and the PDBe-KB Database (19) were applied.

To assess pathogenicity indicators of the variant, the following databases were utilized: NCBI dbSNP (20), ClinVar (VCV003038842.2) (21), ProtVar (22), COSMIC (23), Mutation taster (24), genomeAD browser (25), and VarSome Database (26). To determine the probability of pathogenicity of the tested variant, the subsequent models were used: CADD Score (27), BayesDel Score (28), and EIGEN Score (29).

### 2.8 Statistical analysis

The chi-square test was applied to assess the compliance of the obtained genotype distribution with the Hardy–Weinberg equilibrium. Based on the threshold *p*-value (*p* > 0.05), the variants were involved in the linkage disequilibrium analysis using the Haploview v.4.2 software. To investigate the variants’ associations with the disease the chi-square test was also used or Fisher’s exact test for small group sizes (below 5). The quantitative data were analyzed by the Student’s t-test in case data followed a normal distribution (Shapiro–Wilks test). The Mann–Whitney test for comparison of two subjects groups and Kruskal–Wallis test for comparison of three groups was applied if the data did not follow the normal distribution. These analyses were performed using PQStat 1.8.4 (PQStat Software, Poland) and R software 4.3.2, and all tests were considered significant at *p* value below 0.05. The false discovery rate (FDR) adjusted *p*-values were calculated according to Bonferroni correction.

## 3 Results

A total of 353 IBD patients and 200 individuals from the Polish population were genotyped for both *loci*, c.91G>T and c.-251A>T in the *CXCL8* gene. Pyrosequencing was used to identify the c.-251A>T variant in the analyzed samples. All possible genotypes were detected, with a variant allele frequency of 54% in IBD patients and 51.5% in controls. The KASP method was applied for the stop-gain variant (c.91G>T) in *CXCL8* gene identification. This technique enabled the grouping of samples according to their genotypes. We identified only the heterozygous form of this rare variant, with a frequency of 0.5% in population of Poland and 4.25% in Polish IBD patients. Sanger sequencing with the reverse primer was used to confirm all heterozygotes.

### 3.1 Frequency and allele distribution

The frequency and distribution of all identified genotypes and alleles in IBD patients and the Polish population group are presented in **Table 1**.

**Table 1:** *CXCL8* gene variants distribution and frequency in IBD patients and Polish population subjects.

	Genotype, n (%)			MAF (%)	HWE *p*-value	IBD patients vs. population	
c.-251A>T, rs4073	AA	AT	TT	A		A vs. T	AA vs. AT + TT
IBD patients, n = 353	73 (20.7)	180 (51)	100 (28.3)	46	0.61	*p* = 0.4955 OR 1.10 95%CI [0.86–1.41]	*p* = 0.8246 OR 0.93 95%CI [0.61–1.45]
Population, n = 200	39 (19.5)	116 (58)	45 (22.5)	48.5	0.02		
c.91G>T, rs188378669	GG	GT	TT	T		G *vs.* T	GG *vs.* GT + TT
IBD patients (n=353)	338 (95.8)	15 (4.2)	0 (0)	2.12	0.68	***p* = 0.0121** **(*p*_adj._ = 0.0363)** OR 8.66 95%CI [1.14-65.82]	***p* = 0.0115** **(*p*_adj._ = 0.0345)** OR 8.83 95%CI [1.16-67.36]
Population (n=200)	199 (99.5)	1 (0.5)	0 (0)	0.25	0.25		

HWE, Hardy–Weinberg equilibrium; threshold *p*-value is 0.05; MAF, minor allele frequency; OR, odds ratio; CI, confidence intervals; *p*_adj._, *p*-value after Bonferroni statistical correction; bold font, *p* < 0.05.

The frequency of promoter variant c.-251A>T of the *CXCL8* gene does not differ significantly. It was similar in both patients and control groups and was not much different to the European population in the 1000 Genomes database. For the second variant studied by us, the frequency of the c.91T allele in the *CXCL8* gene was higher in the patient group compared to healthy controls (*p=*0.012) and was also higher than the data from the 1000 Genomes database (which reports a frequency of <1% (30). Therefore, we decided to check whether the differences in frequency would be closely related to CD or UC, comparing both groups of patients (**Table 2**). Performed analysis of c.91G>T presence in CD and UC patients showed no significant differences in allele and genotype distribution.

**Table 2:** Distribution of rs188378669 in *CXCL8* gene among CD and UC patients.

Patients group	Genotype, n (%)			MAF (%)	CD *vs*. UC	
	GG	GT	TT	T	G vs. T	GG vs. GT + TT
**CD** n = 189	182 (96.3)	7 (3.7)	0 (0)	1.9	*p* = 1.0 OR 1.16 95%CI [0.23–5.84]	*p* = 0.8579 OR 1.16 95%CI [0.23–5.96]
**UC** n = 164	157 (95.7)	7 (4.3)	0 (0)	2.1		

MAF, minor allele frequency; OR, odds ratio; CI, confidence intervals; CD, Crohn’s disease; CU, colitis ulcerosa.

### 3.2 Pairwise linkage disequilibrium analysis

**Figure 1:**
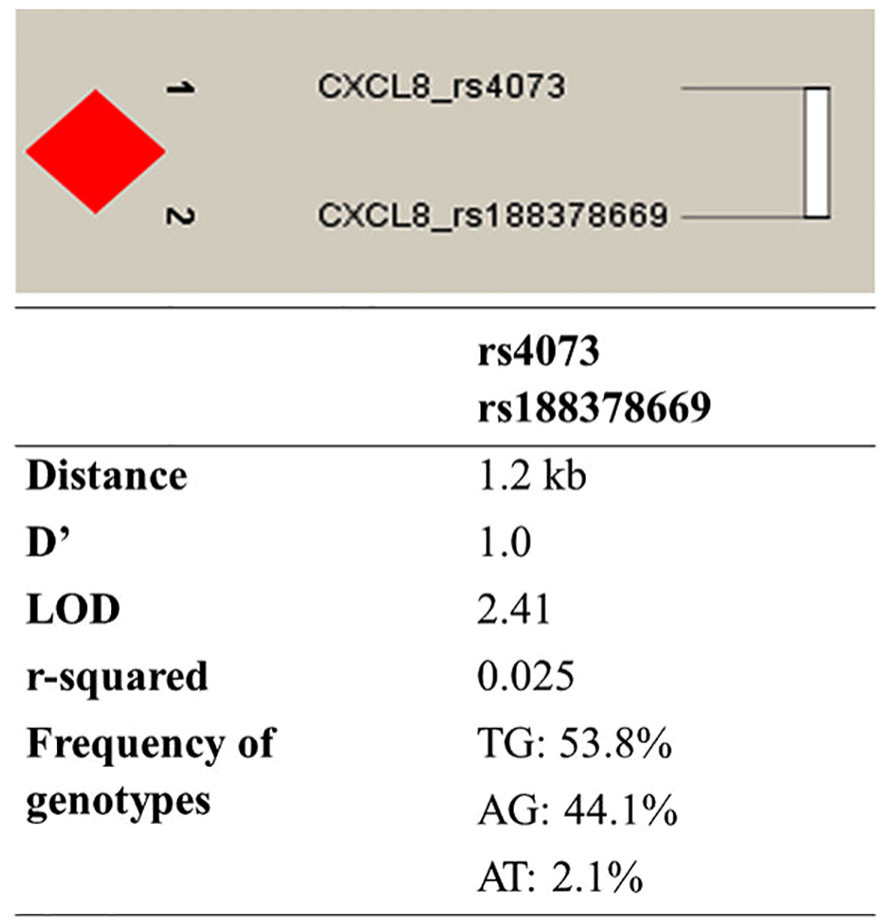
Linkage disequilibrium between analyzed variants in the *CXCL8* gene. Haplotype diagram was prepared with Haploview v.4.2 and show calculated and graphically displayed the absolute value of Lewontin’s measure of linkage disequilibrium, D’ among both studied variants. The intensity of the filled-in box corresponds to the total linkage disequilibrium.

Haplotype analysis was performed to investigate possible linkage disequilibrium between the examined polymorphic changes in the *CXCL8* gene. Results showed that both polymorphisms c.91G>T and c.-251A>T are in strong (D′=1.0) linkage disequilibrium, and three combinations of haplotypes have been observed: TG, AG, and AT, respectively (**Figure 1**).

### 3.3 Variant c.91G>T clinical consequences

For the clinical consequences analysis of the c.91G>T variant, data were collected from the entire group of heterozygous IBD patients (14 subjects) and 23 randomly selected homozygous IBD patients. The characteristics of this group is presented in **Table 3**.

**Table 3:** Subject characteristics included the serum measurement.

Parameter		IBD c.91GT (n = 14)	IBD c.91GG (n = 23)	HC c.91GG (n = 10)	*p*-value	*p*-value adjusted
Sex	F	8 (57.1%)	14 (60.9%)	5 (50.0%)	0.8571	0.8771
	M	6 (42.9%)	9 (39.1%)	5 (50.0%)		
Age, y, mean ± SD; (min–max)		33.3 ± 19.3 (18–55)	40.5 ± 14.2 (24–75)	43.4 ± 6.6 (27–50)	0.3679	0.3679
IBD diagnosis	CD	12 (85.7%)	11 (47.8%)	–	**0.0355** OR 0.16 CI 95% [0.01, 0.97]	**0.0355**
	CU	2 (14.3%)	12 (52.2%)			
Smoker		1 (7.1%)	1 (4.4)	0 (0%)	1.0	1.0
Intestinal location^a^						
Ileal (L1)		2 (14.3%)	0 (0%)	–	0.4783	1.0
Colonic (L2)		4 (28.6%)	2 (8.7%)	–	0.6404	1.0
Ileocolonic (L3)		5 (35.7%)	9 (39.1%)	–	0.193	0.5791
Proctitis (E1)		0 (0.0%)	2 (8.7%)	–	1.0	1.0
Left-sided colitis (E2)		1 (7.1%)	7 (30.4%)	–	1.0	1.0
Extensive colitis (E3)		1 (7.1%)	3 (13.1%)	–	0.5055	1.0
Medication						
Mesalamine		14 (100%)	23 (100%)	–	NA	NA
Corticosteroids		12 (85.7%)	10 (43.5%)	–	**0.0164** OR 7.8, CI 95% [1.41–43.08]	0.0654
Azathioprine		7 (50%)	7 (30.4%)	–	0.3035	1.0
Infliximab/ Adalimumab		9 (64.3%)	8 (34.8%)	–	0.1014	0.4056
Surgical treatment		8 (57.1%)	6 (26.1%)	–	0.0851 OR 3.63, CI 95% [0.75–19.46]	0.3404
Serum IL-8 concentration, pg/ml, mean ± SD (min–max), median		^e^61.7 ± 8.3 (52.3–67.6), 65.4	70.0 ± 19.8 (49.8–124.1), 62.3	51.5 ± 5.9 (44.3–63.0), 52.5	**0.0060** 0.1078^b^, 0.6882^c^, **0.0018**^d^	**0.0060** 0.1616^b^ 0.6882^c^ **0.0055** ^d^

a Disease location were classified according to the Montreal Classification (31); IBD, inflammatory bowel disease; CD, Crohn’s disease; CU, colitis ulcerosa; HC, healthy controls; NA, not analysed.

b IBD c.91GT vs. IBD c.91GG.

c IBD c.91GT vs. HC c.91GG.

d IBD c.91GG vs. HC c.91GG; bold font, *p*-value = 0.05.

e In this group, serum analysis was performed for three persons only.

**Figure 2:**
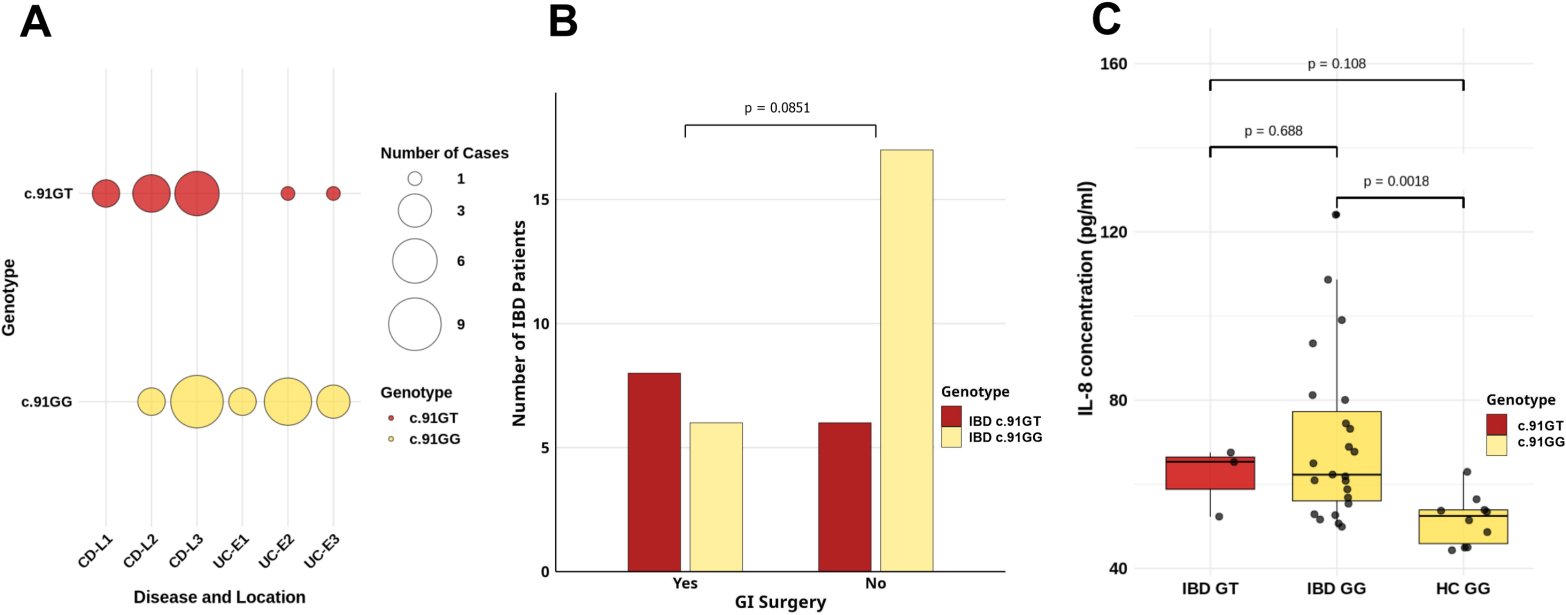
Effect of *CXCL8* gene c.91 genotypes on **(A)** CD and CU location; L1, terminal ileum; L2, colon; L3, ileocolon; E1, ulcerative proctitis, involvement limited to rectum; E2, left-sided ulcerative colitis, involvement limited to a portion of colorectum distal to splenic flexure; E3, extensive ulcerative colitis: involvement extends proximal to splenic flexure. **(B)** GI tract surgical treatment; GI, gastrointestinal, p-value calculated using the Fisher’s exact test before correction, adjusted *p*-value using Bonferroni correction are included in **Table 3**; **(C)** serum IL-8 concentration, median with Q1–Q3, and min.–max. value (whiskers); each point represents a single concentration measurement, *p*-value calculated by non-parametric Mann–Whitney before correction, adjusted *p*-value using Bonferroni correction are included in **Table 3**.

We compared the distribution of GT and GG genotypes in CD and UC locations. Among CD patients with GT genotype, cases with L1, L2, and L3 were found. No homozygotes GG with CD localization in L1 were observed. In turn, among CU carriers of the variant at position c.91, there were only single cases with E2 and E3, and homozygotes GG had E1, E2, and E3. However, no statistically significant differences were found (**Figure 2A**). Regarding GI surgery due to IBD, more patients with the GT genotype underwent surgical treatment compared to GG homozygotes (**Figure 2B**), although this difference was borderline significant before statistically correction (*p* = 0.0851, *p*_adj_. = 0.2389, **Table 3**). Genotype GT also predisposed to the need for treatment with corticosteroids. The odds ratio of using corticosteroids therapy in the GT genotype group are 7.8 times higher than in the homozygous GG group (*p* = 0.0164, *p*_adj_. = 0.0654, **Table 3**).

IL-8 concentration was measured in serum samples from three IBD patients with the heterozygous c.91G>T variant, 23 IBD patients with homozygous GG, and 10 healthy homozygous GG controls. All those 26 IBD patients were in remission state of disease. We observed increased IL-8 concentration in IBD patients with the GG genotype compared to healthy controls with the same genotype at the studied c.91 position of the *CXCL8* gene (*p* = 0.0018, **Figure 2C**; *p*_adj_. = 0.0055, **Table 3**). Meanwhile, the GT heterozygous IBD patients exhibited lower mean IL-8 concentration than GG homozygous IBD patients but higher than GG healthy controls (61.73 vs. 70.02 vs. 51.50, respectively).

### 3.4 Prediction of variant consequences and structure-based functional analysis

To determine the potential consequences of the rs188378669 c.91G>T variant, an *in silico* analysis was conducted, focusing on the conservation of the p.Glu31Ter position as a nonsense variant and its predicted pathogenicity. For this purpose, information regarding the structure of IL-8 was retrieved from AlphaFold. The altered amino acid position in rs188378669, Glu31, receives a pathogenicity score of 0.737 (probability scale: 0, benign; 1, highly pathogenic). This suggests that the Glu31 alteration is likely pathogenic. The structures available in the PDBe-KB (Protein Data Bank in Europe—Knowledge Base) database do not describe (except one entry) the first 29 amino acids of IL-8. These initial amino acids are part of a precursor, and the functional protein begins after this segment. In most cases, protein structure simulations start at the 30th amino acid, as the functional part of the protein begins at this point (32). This fact emphasizes the potential significance of a mutation that causes a stop in protein synthesis at the 31st nucleotide site.

An *in silico* analysis of rs188378669 using NCBI dbSNP, ClinVar (accession, VCV003038842.2), ProtVar, and COSMIC databases indicated that premature protein termination is a predicted consequence of the c.91G>T variant. The MutationTester tool classifies this variant as disease causing.

ProtVar and the gnomAD browser report a CADD score of 35 for this variant, indicating a high likelihood of deleteriousness (CADD score > 29.9 is highly likely to be deleterious). A BayesDel noAF score of 0.588 also indicated (score ranges from −1.29334 to 0.75731) a strongly pathogenic nature of the variant (23). Another source indicating the variant’s pathogenicity is the EIGEN score, which was found to be 0.9117, interpreted as Pathogenic Moderate.

The VarSome database was used to summarize and organize the above data. VarSome assigned the examined variant a high pathogenicity meta-score of 6 (scale: 1, supporting; 2, moderate; 4, strong; 8, very strong). It should be noted, however, that some databases used in the VarSome database, like FATHMM_XF, classify the change as benign, although these represent a minority of the sources (33).

## 4 Discussion

Pathogenesis of IBD, one of the fastest-growing and incurable diseases of modern civilization, despite extensive global research, is still not yet fully elucidated. However, it is considered to be a result of an inappropriate inflammatory response to intestinal microbes in genetically predisposed subjects (34). Given the pivotal role of chemokines in the immune system, we investigated in this study whether the *CXCL8* gene encoding one of the most important of them, IL-8, could be associated with the disease manifestation and the course of the disease. Because IBD is still an incurable disorder associated with major morbidity in Western countries, searching for new IBD-associated loci and therapeutic targets is an extremely urgent challenge currently the more so that it is a complex, polygenic disease. Up to date, over 200 loci associated with IBD have been identified, mainly through genome-wide association studies (GWAS), from which one of the first was the *NOD2* gene (35, 36). Approximately 68% of these loci are common for CD and UC, indicating that these diseases share inflammatory pathways (35). However, the *CXCL8* gene has yet to be identified among them.

In our IBD patients cohort, heterozygotes GT in locus c.91 of the *CXCL8* gene were presented with a frequency of 4.25% compared to 0.5% in the Polish population. We decided to we decided to investigate whether this variant is significantly related either to CD or UC. However, the results showed no association with a specific IBD entity. The presented study has demonstrated for the first time such a correlation of this nonsense substitution c.91G>T in the *CXCL8* gene with the presence of IBD. The results also confirmed our previous observations concerning this variant frequency, based on the NGS study of patients with IBD. It is worth noting that c.91 locus of the *CXCL8* gene has been already associated with early-onset myocardial infarction, hypertension, chronic kidney disease, or endometrial carcinoma (23, 37).

The nucleotide substitution c.91G>T (known as mutation numbered COSV56640974) analyzed in this study has serious consequence—change in the 31st amino acid (Glu) to codon STOP and premature protein chain termination. As a result, a non-functional protein is produced, characterized by reduced stability and receptor-binding affinity, potentially disrupting the proper inflammatory pathway in which IL-8 plays a crucial role (12). Our extensive *in silico* predictive analysis strongly supports the pathogenic nature of the c.91G>T (p.Glu31Ter) variant.

**Figure 3:**
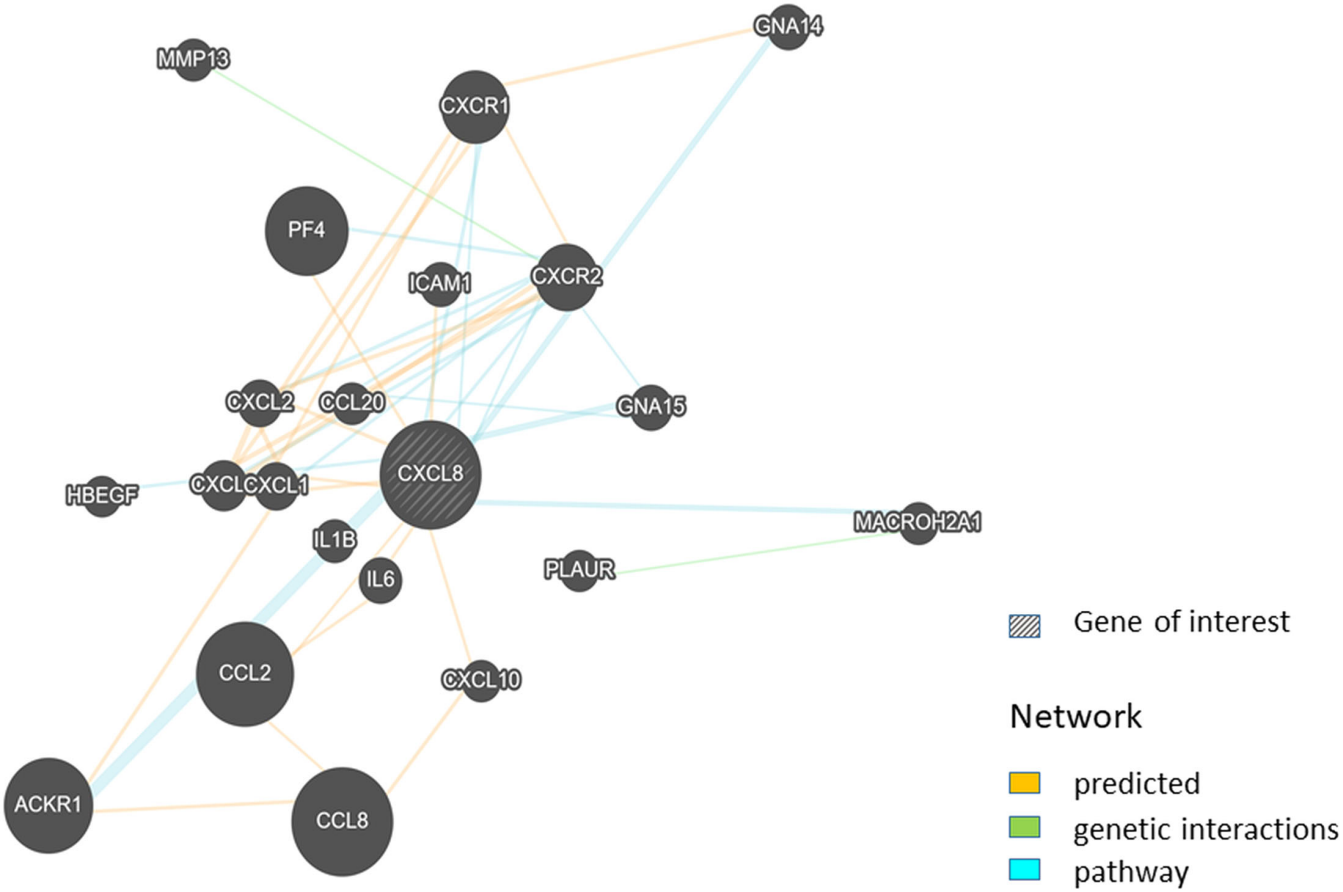
Network analysis of *CXCL8* gene associations. Graph prepared based on BIOGRID database using GeneMANIA tool (44). *CXCL8*, C-X-C motif chemokine ligand 8 (interleukin 8); CCL8, C-C motif chemokine ligand 8; *CCL2*, C-C motif chemokine ligand 2; *ACKR1*, atypical chemokine receptor 1; *PF4*, platelet factor 4; *CXCR1*, C-X-C motif chemokine receptor 1; *CXCR2*, C-X-C motif chemokine receptor 2; *CXCL3*, C-X-C motif chemokine ligand 3; *CXCL1*, C-X-C motif chemokine ligand 1; *CXCL2*, C-X-C motif chemokine ligand 2; *IL6*, interleukin 6; *GNA15*, G protein subunit alpha 15; *GNA14*, G protein subunit alpha 14; *ICAM1*, intercellular adhesion molecule 1; *IL1B*, interleukin 1 beta; *MMP13*, matrix metallopeptidase 13; *MACROH2A1*, macroH2A.1 histone; *CCL20*, C-C motif chemokine ligand 20; *CXCL10*, C-X-C motif chemokine ligand 10; *PLAUR*, plasminogen activator, urokinase receptor; *HBEGF*, heparin binding EGF-like growth factor.

IL-8 activity depends on interaction with the human CXC chemokine receptors CXCR-1 and CXCR-2, atypical chemokine receptor encoded by *ACKR1* gene, and glycosaminoglycans (6). In the case of IBD, IL-8 recruits and activates neutrophils into the lamina propria and epithelium by binding to the CXCR-1/2 receptors (38). The axis IL-8-CXCR-1/2 is crucial in the pathogenesis of UC through multiple signaling pathways, including PI3K/Akt, MAPKs, and NF-κB. There are suggestions that targeted inhibition of this essential axis, IL-8-CXCR-1/2, could be a new therapeutic strategy for the disease (39, 40). Research performed on the DSS-induced mice model revealed that treatment with CXCR-1/2 antagonist improved the colonic condition of mice (6). Chapuy and his research team established a link between monocyte-like CD163− MNPs, IL-12, and IL-1β and the detection of colonic memory IL-8-producing CD4+ T cells, which might all contribute to the pathogenesis of UC but not of CD (41). However, IL-8 is only one of the chemokines produced by inflamed epithelial cells in UC patients, besides C-X-C motif chemokines 1 and 9, and others (42, 43). To better understand the exact role of *CXCL8* in the genes-based network, we created interaction networks using GeneMANIA (**Figure 3**) (44). This network visualizes the link of *CXCL8* with other genes, not only receptor genes but also genes of many abovementioned interleukins, ligands, matrix metalloprotein, intercellular adhesion molecule, and G protein subunits.

Furthermore, recent studies show IL-8 to be a critical factor contributing to tumorigenesis (45). Upregulation of IL-8 was observed at the tumor invasion front in several human cancers. Modulation of IL-8 by CXCR1/2 chemokine receptors promotes various intracellular signaling cascades that lead to tumor-associated inflammation (46). Hence, deciphering the regulatory and signaling function of IL-8 and its further effects may hold prognostic clinical prospects for therapies. Currently, multiple clinical trials are ongoing (6).

Previously, we have also analyzed distribution of this variant c.91G>T in *CXCL8* gene in a group of cystic fibrosis patients from the Polish population, searching for prognostic markers of clinical form of the disease. We found only one heterozygote c.91GT in a group of 55 individuals (47).

Our results here show a borderline association between the GT variant genotype and the need for GI tract surgical treatment, suggesting its relevance to IBD pharmacotherapy effectiveness. Although this study did not explicitly focus on treatment effects, this issue will be addressed in future research. Nevertheless, the observed relationship may indicate the involvement of this variant in the therapy response connected with the disease’s course. Similar association is visible for the GCS treatment; however, further advanced pharmacogenetic investigation are necessary. It should be noted that the mechanism of action of IL-8 is complicated and not obvious. *In vitro* data demonstrate that chemokine IL-8 concentration gradient determines neutrophil motile behaviors. Namely, neutrophils migrate toward increased IL-8 concentration; however, high concentration of IL-8 can repel neutrophils and induce their reverse movement (48, 49).

### 4.1 Limitations of the study

We are also aware of another limitation of this study. The IL-8 serum concentrations were measured only in patients in the remission phase to maintain homogeneous conditions with the control group. However, data from patients during exacerbation would also be valuable and should be included in future studies. Second, we had a limited number of GT genotype patients and even fewer protein studies due to the very low frequency of variant c.91G>T in the *CXCL8* gene. Namely, in the genetic studies of 353 IBD patients, we showed 14 heterozygotes. For serum testing, only three patients simultaneously meets the criterion of no exacerbation and provided a fresh blood sample. With unbalanced groups (with one group with n = 3), the power of the analysis drops from approximately 87.3% to approximately 80.6%.

## 5 Conclusion

The results of our study, presenting new significant associations with the IBD locus in the *CXCL8* gene and defining for the first time IL-8 concentration in IBD p.Glu31Ter carriers, need further confirmation from other populations and functional studies before it may constitute a promising therapeutic value for IBD in the future.

## Data Availability

The original contributions presented in the study are included in the article. Further inquiries can be directed to the corresponding authors.
