# Corrigendum: Is a rare *CXCL8* gene variant a new possible cause or course factor of inflammatory bowel disease?

**DOI:** 10.3389/fimmu.2025.1599125

**Published:** 2025-04-11

**Authors:** Marcin Gabryel, Oliwia Zakerska-Banaszak, Karolina Ladziak, Katarzyna Anna Hubert, Alina Baturo, Joanna Suszynska-Zajczyk, Magdalena Hryhorowicz, Agnieszka Dobrowolska, Marzena Skrzypczak-Zielinska

**Affiliations:** ^1^ Department of Gastroenterology, Dietetics and Internal Medicine, Poznan University of Medical Sciences, Poznan, Poland; ^2^ Institute of Human Genetics, Polish Academy of Sciences, Poznan, Poland; ^3^ Department of Biochemistry and Biotechnology, Poznan University of Life Sciences, Poznan, Poland

**Keywords:** CXCL8 gene, inflammatory bowel disease (IBD), inflammation, interleukin 8, genetic variants, Crohn’s disease, colitis ulcerosa

In the published article, there was an error in the article title. Instead of “Is a rare *CXCL8* gene variant a new possible cause or curse factor of inflammatory bowel disease?”, it should be “Is a rare *CXCL8* gene variant a new possible cause or course factor of inflammatory bowel disease?”.

The authors apologize for this error and state that this does not change the scientific conclusions of the article in any way. The original article has been updated.

